# Government-led scale-up of task-shifted mental health services in Lagos State, Nigeria: a mixed-methods descriptive–explanatory case study

**DOI:** 10.1093/heapol/czag033

**Published:** 2026-03-10

**Authors:** Abiodun O Adewuya, Bolanle Ola, Olurotimi Coker, Olayinka Atilola, Olushola Olibamoyo, Azizat Lebimoyo, Olabisi Oladipo

**Affiliations:** Department of Behavioural Medicine, Lagos State University College of Medicine, 1-5 Oba Akinjobi Way, Ikeja 100010, Lagos, Nigeria; Public Mental Health Department, Centre for Mental Health Research and Initiative, 3rd Floor, Old Glass House, LASUCOM Complex, Ikeja 10010, Lagos, Nigeria; Department of Behavioural Medicine, Lagos State University College of Medicine, 1-5 Oba Akinjobi Way, Ikeja 100010, Lagos, Nigeria; Department of Behavioural Medicine, Lagos State University College of Medicine, 1-5 Oba Akinjobi Way, Ikeja 100010, Lagos, Nigeria; Department of Behavioural Medicine, Lagos State University College of Medicine, 1-5 Oba Akinjobi Way, Ikeja 100010, Lagos, Nigeria; Department of Behavioural Medicine, Lagos State University College of Medicine, 1-5 Oba Akinjobi Way, Ikeja 100010, Lagos, Nigeria; Department of Behavioural Medicine, Lagos State University College of Medicine, 1-5 Oba Akinjobi Way, Ikeja 100010, Lagos, Nigeria; Public Mental Health Department, Centre for Mental Health Research and Initiative, 3rd Floor, Old Glass House, LASUCOM Complex, Ikeja 10010, Lagos, Nigeria

**Keywords:** mental health integration, health systems strengthening, task-shifting, digital supervision, Nigeria

## Abstract

Mental health conditions remain a leading contributor to global disability; however, treatment coverage in low- and middle-income countries (LMICs) stays below 20%; in Nigeria, services are underfunded and largely excluded from primary health care (PHC). This study documents the institutional processes through which the Transition-to-Scale phase of the Mental Health in Primary Care (MeHPriC) initiative scaled up task-shifted mental health services across Lagos State, Nigeria. A retrospective, mixed-methods descriptive–explanatory case study was conducted across 57 PHCs and five general hospitals. The intervention delivered care for five priority mental, neurological, and substance use conditions using the Mental Health Gap Action Programme (mhGAP) framework; 890 health workers were trained, comprising 400 Community Health Extension Workers (CHEWs), 250 nurses, 150 medical officers, 85 lay counsellors, and 5 district psychiatrists, under structured district-level supervision. Data from service registers, supervision checklists, stock audits, provider and client surveys, key informant interviews, focus groups, and policy documents were analysed using descriptive statistics and hybrid deductive–inductive thematic coding organized around the World Health Organization (WHO) Health System Building Blocks and selected Consolidated Framework for Implementation Research (CFIR) constructs. The initiative was associated with institutional changes across governance (establishment of a Mental Health Desk and a multisectoral Stakeholders Council), workforce supervision (fidelity rising from 13% to 92.3% of facilities conducting weekly case reviews), medicines (six psychotropic medications added to the Essential Medicines List; stockouts reduced by 42%), financing (₦75 million allocated through routine government budgeting), service delivery (64 107 clients screened and 9138 initiated on treatment), and health information systems. Interpreted as incremental strengthening within the mental health subsystem, these findings reinforce the feasibility of mhGAP-aligned task-shifted care when supported by structured supervision and governance, while persistent fiscal and operational constraints underscore the fragility of institutional gains.

Key messages

- Although mental health programmes in LMICs have frequently demonstrated pilot feasibility, far fewer studies have documented the institutional processes through which task-shifted mental health services become progressively embedded within routine government systems during scale-up. This paper provides such an account from a government-led initiative in Lagos State, Nigeria.
- The MeHPriC Transition-to-Scale phase embedded care for five priority mental, neurological, and substance use disorders into 57 primary health centres and five general hospitals. Nonspecialist cadres, particularly community health extension workers and nurses, assumed primary frontline responsibilities under structured supervision by district psychiatrists, supported by digitally mediated (WhatsApp) consultation networks.
- Over the implementation period, measurable institutional changes were observed across governance, supervision, medicines procurement, financing, service delivery, and health information systems. These are interpreted as incremental strengthening within the mental health subsystem rather than as evidence of system-wide reform, a distinction that carries important implications for how scale-up outcomes are framed and evaluated.
- Digital supervision platforms and participatory governance mechanisms appear to have played enabling roles in sustaining clinical quality and policy responsiveness during scale-up, although their long-term durability under changing political and fiscal conditions remains to be assessed.

## Introduction

### Global burden of mental health and the scale-up challenge

Mental, neurological, and substance use (MNS) disorders constitute a substantial global public health challenge, collectively accounting for approximately 13% of the global burden of disease measured in disability-adjusted life years (GBD 2019 Mental Disorders Collaborators 2022). Depressive disorders are projected to become one of the leading contributors to disease burden worldwide by 2030 (Fan et al. 2025), suicide claims nearly 800 000 lives annually (Naghavi 2019), and epilepsy affects more than 50 million people globally, approximately 80% of whom reside in low- and middle-income countries (LMICs) (Singh and Sander 2020). Despite this burden, more than 80% of people living with mental health conditions in LMICs receive no formal treatment, reflecting persistent structural, financial, and sociocultural barriers to access (Rathod et al. 2017).

Over the past two decades, task-shifting strategies (redistributing selected clinical functions from specialist providers to trained nonspecialist health workers) have emerged as a pragmatic response to workforce scarcity and service inequities (Bolton et al. 2023, Ndetei et al. 2023). The World Health Organization's Mental Health Gap Action Programme (mhGAP) has provided a standardized framework for scaling evidence-based interventions for priority MNS conditions within primary health care systems (World Health Organization 2008). Large multicountry initiatives, including the Programme for Improving Mental Health Care (PRIME) and the Emerging Mental Health Systems in LMICs (EMERALD) programme, have demonstrated the clinical feasibility and acceptability of integrating mental health into routine primary care platforms (Lund et al. 2012, Semrau et al. 2015).

Much of this empirical literature, however, remains anchored in demonstration pilots or time-limited externally funded projects. Evidence on the institutionalization of governance arrangements, financing mechanisms, supervision structures, supply chains, and health information systems during scale-up remains fragmented. Parallel debates within global health, particularly in relation to vertically funded human immunodeficiency virus (HIV) programmes, have highlighted both the potential and the limits of disease-specific initiatives as vehicles for broader system strengthening (Sheikh et al. 2011). Within mental health specifically, there is a relative paucity of longitudinal, system-embedded case studies documenting how scale-up processes interact with public-sector governance and administrative architectures over time.

### The Nigerian and Lagos mental health context

Nigeria exemplifies the challenges of translating technical feasibility into sustained institutional integration. With a population exceeding 200 million, the country carries a substantial burden of mental disorders: the point prevalence of major depressive disorder is estimated at 5.6%, anxiety disorders affect approximately 3.4% of the population (Gureje et al. 2010, Adewuya et al. 2018), and the national mental health treatment gap exceeds 83%, which is among the highest globally (Gureje and Lasebikan 2006). Services have historically been concentrated in tertiary psychiatric facilities, with chronic under-financing (consistently below 1% of state health budgets), workforce shortages, weak supply chains, and minimal integration into primary health care collectively constraining service expansion (Abdulmalik et al. 2019). Sociocultural barriers, including stigma and explanatory models that prioritize spiritual or traditional healing, further shape help-seeking behaviour (Rathod et al. 2017).

Lagos State, a megacity of more than 20 million residents, operates a decentralized public health system governed by the Ministry of Health (MoH), the Primary Health Care Board (PHCB), and the Health Service Commission (HSC). Despite comparatively stronger administrative capacity and fiscal resources than many Nigerian states, mental health services historically remained marginal within routine service delivery and budgeting processes, an institutional landscape that presented both opportunities and constraints for embedding scale-up within existing government systems.

### The MeHPriC programme and prior evidence

The Mental Health in Primary Care (MeHPriC) initiative was developed to address the mental health treatment gap in Lagos through a task-shifting model aligned with the mhGAP framework. The pilot phase, launched in 2013 with support from Grand Challenges Canada, demonstrated the feasibility of training nonspecialist providers to deliver assessment, psychoeducation, pharmacological treatment, and referral for priority MNS conditions across 15 primary health centres (Adewuya et al. 2019a, 2019b).

Building on this experience, the Transition-to-Scale (TTS) phase sought to expand service coverage across multiple districts while progressively embedding key functions within government systems. This phase emphasized structured supervision through district psychiatrists, digitally mediated clinical support, integration of psychotropic medicines into public procurement, formal governance mechanisms within the MoH, and alignment of financing with routine public-sector workflows. Prior publications have documented mhGAP training outcomes (Adewuya et al. 2025a) and contextual determinants of sustainability (Adewuya et al. 2025b); broader comparative analyses have examined scaling up integrated mental health across multiple LMIC contexts (Petersen et al. 2019). Fewer studies, however, have examined how interventions interact with administrative, regulatory, and managerial systems at the governance level during scale-up or how ownership evolves beyond project-driven implementation. The present study, therefore, focuses on the institutional and governance processes through which scale-up was operationalized, rather than on clinical effectiveness.

### Conceptual framing: system strengthening and task shifting

This study adopts a framing centred on system strengthening and institutional embedding within the mental health subsystem, drawing on evidence that targeted investments may strengthen selected system functions without necessarily generating cross-sectoral spillover or durable institutional transformation (Sheikh et al. 2011). The analysis is organized using the WHO Health System Building Blocks framework, which distinguishes six core domains: governance, health workforce, service delivery, financing, access to essential medicines, and health information systems (World Health Organization 2007). To interpret the contextual and procedural determinants shaping implementation, selected constructs from the Consolidated Framework for Implementation Research (CFIR) are employed as an interpretive lens, focusing on intervention characteristics, organizational context, leadership, and implementation processes (Damschroder et al. 2009). Complementing these, the health system “hardware and software” perspective foregrounds the interaction between formal institutional arrangements (policies, budgets, and infrastructure) and relational dynamics, including trust, professional norms, and informal leadership (Sheikh et al. 2011). Together, these perspectives enable examination of what institutional changes occurred, how they were enacted, and under what enabling or constraining conditions.

Task shifting is understood here as the redistribution of selected mental health care functions from specialist psychiatrists to trained nonspecialist cadres within primary and secondary care, supported by structured supervision and referral mechanisms (World Health Organization 2008). The inclusion of neurological conditions such as epilepsy reflects the mhGAP classification of priority MNS disorders while recognizing ongoing debates regarding appropriate task allocation and clinical boundaries in resource-constrained settings (Singh and Sander 2020).

### Study objectives

This study has three objectives. First is to describe how the TTS phase of MeHPriC expanded and institutionalized task-shifted mental health services across governance, workforce, supervision, medicines, financing, service delivery, and information systems. Second is to identify selected enabling and constraining factors as observed by participants; and third is to generate empirically grounded lessons for government-led scale-up of integrated mental health services in comparable LMIC contexts.

## Methods

### Study design and case definition

This study employed a retrospective, embedded, mixed-methods descriptive–explanatory case study design to examine the institutional embedding of a government-led mental health scale-up initiative in Lagos State, Nigeria. Case study methodology was selected because it allows an in-depth examination of complex interventions operating within real-world institutional contexts where boundaries between the intervention and the system are not sharply delineated (Yin 2018). The descriptive–explanatory orientation reflects the study’s dual emphasis on documenting observed institutional changes and interpreting the contextual and organizational conditions under which they unfolded.

The primary unit of analysis was the Lagos State public-sector mental health subsystem during the TTS phase. Embedded subunits included clusters of linked primary health centres and general hospitals, district-level supervisory structures, and state-level governance and financing mechanisms. The study was not designed as a comparative evaluation between clusters nor did it include a formal control group; inferences are therefore limited to plausible associations and implementation processes rather than causal effects.

### Conceptual and analytical framework

**Figure 1:**
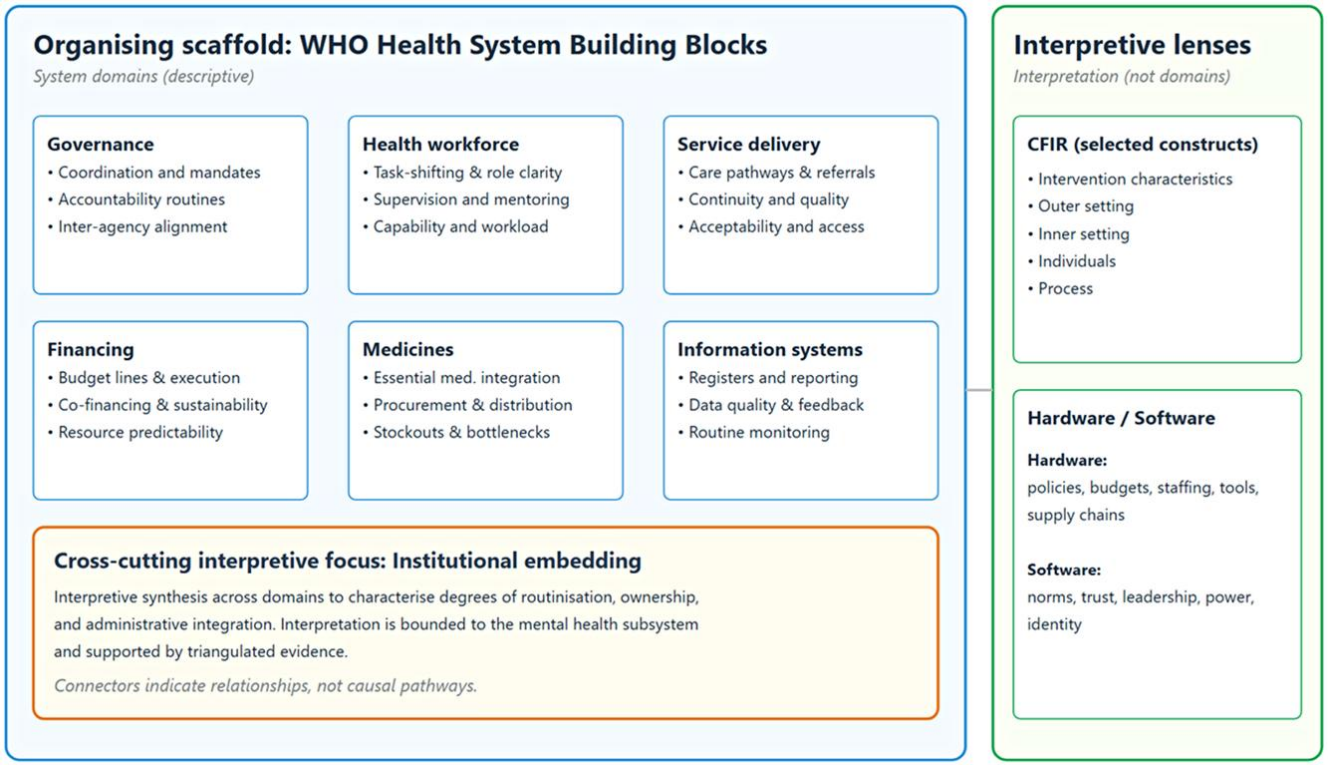
Analytic organizing and interpretive lenses used to examine institutional embedding of mental health services.

The analysis was guided by three complementary frameworks introduced in Section 1.4, whose relationship is illustrated in Fig. 1. The WHO Health System Building Blocks framework structured the Results section across six domains: governance, health workforce, service delivery, financing, access to essential medicines, and health information systems (World Health Organization 2007). CFIR was applied as an interpretive lens to explore contextual and processual determinants of implementation, attending to intervention characteristics, outer and inner setting features, individual-level factors, and implementation processes (Damschroder et al. 2009). The hardware–software perspective examined how formal institutional arrangements interacted with relational and normative dimensions of trust, professional norms, and power dynamics during scale-up (Sheikh et al. 2011). These frameworks guided sampling, data collection, coding, and synthesis rather than functioning as a causal pathway model.

### Study setting and intervention overview

The study was conducted in Lagos State, Nigeria, a metropolitan region with a decentralized public health system administered by the MoH, the PHCB, and the HSC. The MeHPriC TTS phase was implemented between mid-2017 and mid-2019 across 57 comprehensive primary health centres and five general hospitals spanning all five administrative divisions of the state, with each primary health centre linked to a general hospital serving as a supervisory and referral hub. An extended monitoring period continued until December 2019 to assess the durability of institutional changes.

The intervention was designed as a multi-component public-sector programme embedding mental health services within routine primary and secondary care through a task-shifting model aligned with the mhGAP Intervention Guide (World Health Organization 2008). Priority conditions included depression, psychosis, epilepsy, substance use disorders, and suicide risk. Core components comprised in-service training of non-specialist cadres, structured supervision by district psychiatrists, digital supervision via WhatsApp groups, integration of essential psychotropic medicines into public procurement, formal governance mechanisms within the MoH, routine budget alignment, and introduction of mental health indicators into facility registers and pilot health information platforms. Detailed operational features are described in the Results section and Supplementary Materials.

### Data sources and sampling

Multiple quantitative and qualitative data sources were used to enable triangulation across domains. Quantitative sources included routine service registers, supervision checklists, medicine stock audits, and structured provider and client surveys. Qualitative sources comprised key informant interviews, focus group discussions, policy documents, administrative records, and anonymized WhatsApp supervision logs.

Facility-level quantitative data were extracted from all 57 participating primary health centres and five general hospitals. Survey samples were drawn from trained providers (*n* = 726; response rate 82%) and service users attending participating facilities (*n* = 2380). Qualitative participants were purposively selected to ensure representation across administrative levels, professional cadres, and geographic divisions (key informant interviews: *n* = 40; focus group discussions: *n* = 6). A detailed matrix of data sources, indicators, sampling frames, tools, and response rates is provided in Supplementary Table S1.

### Data collection procedures

Quantitative data were extracted from facility registers, supervision tools, and stock audit forms using standardized templates. Data collectors were trained in abstraction procedures, quality checks, and confidentiality protocols, with routine checks conducted to identify missing or inconsistent entries. Surveys were administered using structured questionnaires.

Qualitative interviews and focus groups followed semistructured guides aligned with the analytical framework, exploring implementation experiences, governance processes, supervision dynamics, resource constraints, and perceived sustainability. Interviews were audio-recorded with consent, transcribed verbatim, and anonymized. WhatsApp supervision data were extracted from group archives over defined time windows with identifiers removed, capturing clinical query volumes, response times, thematic discussion categories, and interaction patterns, under institutional approvals and data protection protocols.

### Data analysis and integration

Quantitative data were analysed using descriptive statistics to summarize trends in service utilization, supervision coverage, medicine availability, financing indicators, and reporting completeness. No inferential analyses were conducted, given the descriptive orientation and absence of a comparison group.

Qualitative data were analysed thematically using a hybrid deductive–inductive coding approach, with initial categories informed by the Building Blocks domains and CFIR constructs and inductive refinement applied to capture emergent themes. Coding was conducted iteratively, supported by analytic memos. Intercoder reliability was assessed on a subsample of transcripts, yielding a Cohen’s kappa coefficient above 0.80.

Integration followed a convergent mixed-methods approach, with findings compared and synthesized within each analytical domain to identify convergent, complementary, or discrepant patterns. Joint displays were used during internal analysis meetings to facilitate triangulation. Attribution was approached cautiously, with findings interpreted as plausible associations rather than causal effects.

### Ethics, reflexivity, and positionality

Ethical approval was obtained from the Health Research and Ethics Committee of the authors’ institution. All participants provided informed consent, and data were anonymized and stored securely in accordance with institutional policies.

Given that members of the research team were involved in programme implementation, reflexive practices were incorporated to mitigate potential bias, including analytic audit trails, multiple-analyst coding and interpretation, and triangulation across independent data sources. A validation workshop convened by the Lagos State Ministry of Health in November 2019, involving representatives from the PHCB, district hospitals, implementing partners, and community leaders, provided an additional check on interpretations.

## Results

### Governance and leadership

At the outset of the TTS phase, Lagos State had no dedicated mental health coordination structure within either the Ministry of Health or the Primary Health Care Board, and decision-making related to mental health occurred on an *ad hoc* basis. To address this gap, MeHPriC engaged in a sustained process of institutional advocacy, working with senior officials within the Directorate of Public Health to identify an appropriate organizational home for mental health coordination, drafting terms of reference for the proposed unit, and providing technical support for the recruitment and orientation of staffing positions. This facilitation process, conducted over several months in 2016–2017, resulted in the formal establishment of a Mental Health Desk within the Directorate of Public Health at the MoH in early 2017. The unit was staffed by two full-time officers, appointed and remunerated through the state civil service budget, and given a formal mandate for mental health planning, monitoring, and inter-agency coordination. Its creation marked the first time that mental health had been structurally embedded as a defined function within the Lagos State public health system.

Concurrently, the Mental Health Stakeholders Council, initially a loosely organized forum, was revitalized and formalized as a quarterly convening platform. The Council brought together representatives from the MoH, PHCB, general hospitals, universities, civil society, and user groups. Analysis of meeting minutes from six Council sessions showed that key policy decisions, including revision of the Essential Medicines List to include psychotropic medications and the adoption of new supervisory structures, were deliberated and endorsed through this platform. Interviews with senior officials emphasized that the Council enhanced transparency, fostered policy ownership, and created feedback loops that enabled adaptive course correction during implementation. One policymaker described the Council as:

‘*a space where everyone could see the data and agree on what to fix first’*

***—Policy-level respondent***

Further accounts from policy-level respondents elaborated how the formal positioning of the Mental Health Desk altered institutional legitimacy while remaining dependent on individual agency:

‘*At the beginning, mental health activities were mostly driven by the project team, so other departments did not really see it as part of routine work. Once the Mental Health Desk was formally created inside the Ministry and staff were posted there, mental health started appearing in planning meetings and internal memos. It gave the programme more legitimacy, even though progress still depends on committed individuals pushing the agenda.’*

***—Policy-level respondent***

Monitoring data indicated that both the Mental Health Desk and the Stakeholders Council continued to function beyond the formal project period, with ongoing coordination activities and regular meetings reported up to December 2019, as shown in Table 1. Despite these advances, respondents noted variable engagement across departments and continued dependence on leadership continuity, indicating that institutional embedding remained uneven.

**Table 1:** Governance and institutional integration reforms during the transition-to-scale phase.

Institutional mechanism	Year	Responsible unit	Founding source	Evidence source	Status at endline	Attribution
Establishment of Mental Health Desk	2017	Lagos State MoH (Directorate of Public Health)	Initially project-supported; later absorbed into government	MoH circulars; KIIs	Institutionalized	Direct facilitation
Appointment of two desk officers	2017	MoH	Government payroll	HR records; KIIs	Institutionalized	Direct facilitation
Mental Health Stakeholders Council	2018	MoH; PHCB; HSC	Government	TORs; meeting minutes	Functional but variably active	Enabling
Appointment of district psychiatrists (*n* = 5)	2017–2018	Health Service Commission	Government	Job descriptions; payroll	Fully integrated into civil service	Direct facilitation
Inclusion in annual operational plans	2019	MoH Planning Unit	Government	Planning documents	Partial integration	Contextual
Inclusion in MoH-approved CME	2019	MoH	Government	Training calendars	Integrated	Enabling

Attribution categories distinguish direct facilitation (active project engagement), enabling (creating conditions), and contextual (alignment with existing processes).

MoH, Ministry of Health; PHCB, Primary Health Care Board; HSC, Health Service Commission; CME, Continuing Medical Education; KIIs, Key Informant Interviews.

### Health workforce, task shifting, and supervision

Before MeHPriC, there was no formal supervision structure for mental health at the primary health care level in Lagos State; psychiatrists were largely confined to tertiary institutions, and PHC workers had minimal access to ongoing clinical mentorship. During the TTS phase, five district psychiatrists were recruited into the Lagos State civil service and deployed to the five general hospitals designated as regional supervisory hubs. Each psychiatrist was responsible for a cluster of approximately 10–15 primary health centres, providing both in-person and remote clinical oversight.

In total, 890 public-sector health workers were trained under the TTS initiative. The training cohort comprised 400 CHEWs, 250 nurses, 150 medical officers, 85 lay counsellors, and 5 district psychiatrists. Training completion rates exceeded 95%, and provider survey data indicated substantial gains in knowledge and confidence in managing MNS conditions. More than 70% of respondents reported feeling “much more confident” or “confident” in independently managing common MNS presentations following training and supervision.

The workforce strategy prioritized redistribution of selected mental health tasks from specialist psychiatrists to nonspecialist cadres, particularly nurses and CHEWs, consistent with mhGAP guidance. Task shifting was operationalized primarily at the level of nonphysician providers, who assumed frontline screening, psychoeducation, follow-up, and referral responsibilities. Medical officers participated in training and supervision but were not the primary focus of task-shifting activities; their role was oriented towards diagnosis, medication initiation, and referral coordination within mixed-cadre teams. This distinction is important because it clarifies the actual minimum level at which task shifting was operationalized, namely among CHEWs and nurses rather than among doctors, and raises questions about the feasibility of extending similar arrangements to lay health workers in settings with even greater workforce constraints.

**Figure 2:**
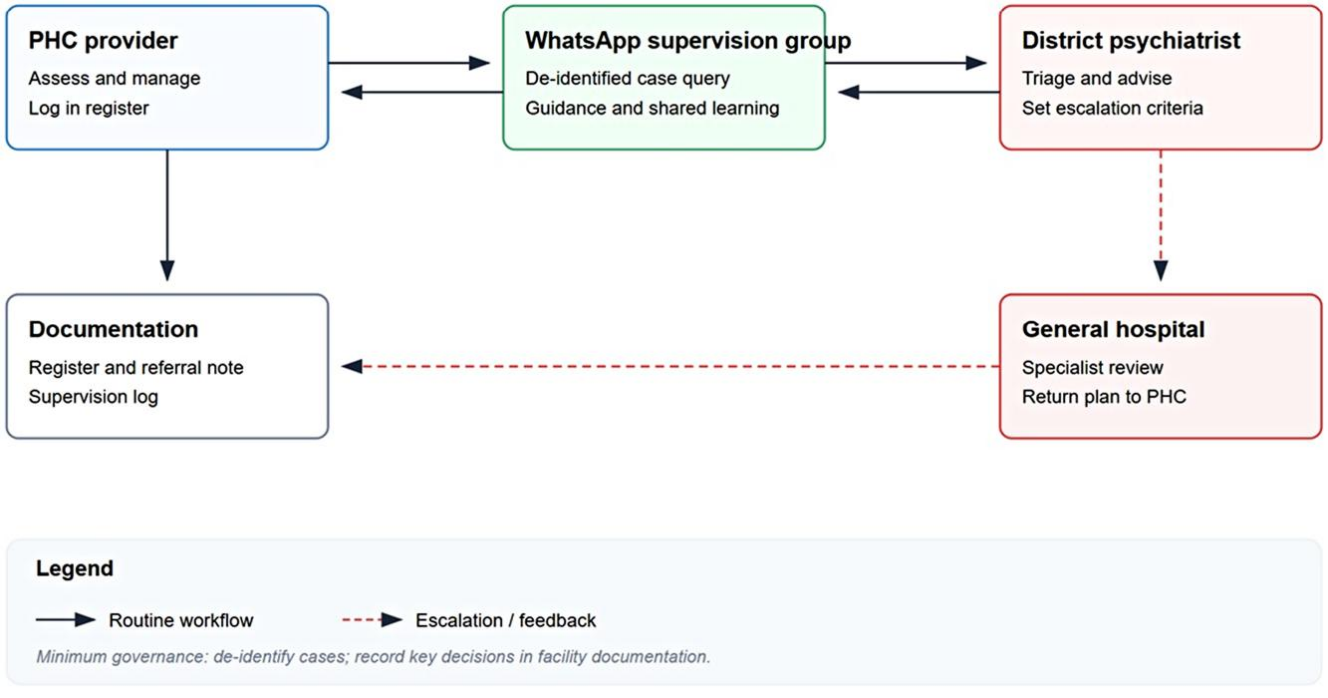
Digital supervision and escalation pathway.

One of the most significant workforce innovations was the development of WhatsApp-based supervision groups. Each district established a dedicated group comprising PHC clinicians, the district psychiatrist, and general hospital support staff; across all five districts, over 500 PHC workers participated. The supervision structure featured a tiered digital pathway for routine support and escalation of complex queries, as illustrated in Fig. 2. Analysis of digital logs from a six-month monitoring window documented an average of 34 clinical queries per district per month, with a median response time of under 4 hours. Queries most commonly concerned diagnostic clarification, side-effect management, and referral pathways. Survey data showed that 90.8% of trained health workers rated WhatsApp supervision as ‘very helpful’ or ‘essential’ to their clinical confidence.

Providers described how this form of digital supervision supported experiential learning and confidence development:

‘*Before the training and WhatsApp support, we were not confident managing mental health cases and usually referred immediately. Now we assess first and post difficult cases on the group, and the psychiatrist guides us on what to do. Over time, you start recognising patterns and managing simpler cases yourself, and you also feel supported rather than isolated.’*

***—Primary care provider***

Another provider emphasized how the platform lowered barriers to consultation:

‘*You know someone will answer you quickly, so you are not afraid to ask questions.’*

***—Primary care provider***

Supervision fidelity improved markedly over the course of the TTS phase. By the end of the implementation period, 92.3% of PHCs were conducting weekly case reviews using standardized job aids, compared with 13% at baseline, and supervisory audit checklists rated 87% of participating PHCs as having ‘good’ or ‘excellent’ documentation practices (Table 2). Respondents emphasized that the combination of scheduled physical visits with continuous digital engagement enabled more frequent touchpoints and rapid troubleshooting than had previously been possible within existing human resource constraints.

**Table 2:** Health workforce, task shifting, and supervision coverage.

Cadre	Number trained	Primary clinical roles	Supervision modality	Minimum supervision expectation	Funding source
CHEWs	400	Screening; psychoeducation; follow-up; referral	WhatsApp + on-site	Monthly group supervision plus ad-hoc digital support	Government
Nurses	250	Assessment; medication administration; follow-up	WhatsApp + on-site	Monthly group supervision	Government
Medical officers	150	Diagnosis; medication initiation; referral coordination	On-site + WhatsApp	Quarterly on-site supervision	Government
Lay counsellors	85	Psychoeducation; adherence support	WhatsApp	Monthly review	Project
Pharmacy technicians	5 (per district)	Supply oversight; stock reporting	On-site	Quarterly audit	Government
District psychiatrists	5	Clinical supervision; escalation; mentoring	On-site + WhatsApp moderation	Bi-weekly district rotation	Government

Total trained = 890. Pharmacy technicians and clerical officers also received orientation but were not primary clinical task-shifting targets.

CHEWs, Community Health Extension Workers.

### Access to essential medicines

A baseline assessment in late 2016 found that psychotropic medications were almost entirely absent from PHC formularies across Lagos State. None of the state’s 319 PHCs had consistent stocks of antidepressants, antipsychotics, or antiepileptic drugs, and psychotropics were excluded from the Lagos State Essential Medicines List, making it administratively impossible for the PHCB to plan or budget for their routine procurement and distribution.

During the TTS phase, acting on recommendations from the Stakeholders Council, the MoH formally revised the Essential Medicines List to include six psychotropic medications: amitriptyline, haloperidol, chlorpromazine, diazepam, phenobarbital, and carbamazepine. This change allowed psychotropics to be incorporated into state procurement processes for the first time. The policy revision was followed by the first state-funded procurement of psychotropic medicines in 2017, with supplies warehoused at district hospitals and redistributed to PHCs based on reported consumption and stock levels.

Stock audit data demonstrated a 42% reduction in psychotropic stockouts across the 57 participating PHCs during the implementation period. At baseline, 71% of PHCs reported zero availability of psychotropics; by early 2018, 59% maintained consistent stocks of at least three essential psychotropic medicines (Table 3). Interviews with pharmacists and facility managers highlighted the role of general hospitals as intermediaries in buffering supply shocks and supporting last-mile distribution. The introduction of a quarterly reporting system improved visibility of stock levels and enabled programme managers to respond more quickly to emerging gaps. Nonetheless, persistent stockouts were reported in some riverine and periurban areas, underscoring ongoing weaknesses in logistics and forecasting.

**Table 3:** Availability of essential psychotropic medicines in participating primary health centres.

Medicine	Included in revised EML	Baseline availability	Endline consistent stock (%)	Stockout reduction (%)	Key operational bottleneck
Amitriptyline	Yes	<40%	65	45	Forecasting delays
Haloperidol	Yes	40%–50%	59	38	Distribution lag
Chlorpromazine	Yes	Low	61	40	Procurement timing
Phenobarbital	Yes	Low	54	36	Storage constraints
Carbamazepine	Yes	30%–40%	58	39	Redistribution
Diazepam	Yes	50%–60%	62	42	Storage constraints
Overall	NA	71% zero availability	59% with ≥3 medicines	42	Systemic supply fragility

Availability reflects the proportion of facilities with uninterrupted stock during the reporting period. Stockout days were averaged across facilities.

EML, Essential Medicines List.

Qualitative accounts captured the perceived trade-offs between sustainability and predictability:

‘*When medicines were supplied directly by the project, it was more predictable, but we worried about sustainability. Now they are in the state procurement system, which is better for long-term continuity, but sometimes delays happen, and stock can run low. So integration has improved ownership, even though operational challenges remain.’*

***—Facility manager***

### Health financing and budget integration

At the start of the TTS phase, mental health had no dedicated line item in the PHCB operational budget, and activities during the earlier pilot phase had been fully donor-funded and largely managed outside government financial systems. One of the strategic aims of the TTS initiative was therefore to embed mental health within existing public financing architecture as a step towards sustainability.

During implementation, the MoH secured an allocation of ₦75 million as a dedicated budget for mental health integration. This funding, approved through the Ministry’s standard Medium-Term Sector Strategy process and integrated into its annual budget, covered training refreshers, psychotropic medicine procurement, and logistics support (Table 4). Additional resources were leveraged from Lagos State’s health basket fund, enabling some facilities to procure consumables required for mental health consultations. Interviews with finance officials indicated that the combination of local evidence from MeHPriC and advocacy through the Stakeholders Council was perceived as instrumental in obtaining this allocation, although they also acknowledged the influence of broader political and fiscal priorities.

**Table 4:** Health financing integration and budget trajectory for mental health activities.

Financial indicator	Funding source	Execution rate (%)	Covered activities	Sustainability risk
Dedicated mental health budget (₦75 million)	Government	60–70	Training refreshers; supervision; logistics	Moderate
Psychotropic medicines procurement	Government	65–75	Six EML medicines; distribution	Moderate
Training and refresher courses	Blended (government + project)	50–60	Provider training; competency updates	High
Digital supervision support	Project	85–95	Data costs; group moderation	High
Programme-based budgeting	Not yet established	NA	Pending full institutionalisation	High

Execution rate reflects the proportion of allocated budget that was disbursed. Sustainability risk reflects a qualitative assessment of funding continuity based on stakeholder interviews.

Despite these gains, full financial institutionalization remained incomplete. While mental health activities were incorporated into departmental workplans, mental health was not yet embedded as a standalone programme within the state’s programme-based budgeting framework. Staff within the PHCB expressed concern that, in the absence of a fully recurrent budget line and explicit programme codes, allocations could be vulnerable to future fiscal pressures or leadership changes.

Stakeholders articulated this tension clearly:

‘*Having a budget line for mental health means it is now recognized in formal planning, but release of funds still depends on overall government finances and competing priorities. We often need to follow up closely to ensure activities are funded as planned. So the system has improved, but it is not yet fully predictable.’*

***—Senior administrator***

Nevertheless, the shift from exclusively donor-funded activities to partial state cofinancing represented a significant step towards governmental ownership of mental health integration.

### Service delivery and clinical protocol integration

The TTS phase was associated with a substantial expansion in service delivery for MNS conditions at PHC level. Over a 12-month period, 64 107 clients were screened for MNS conditions across the 57 PHCs, and 9138 were initiated on treatment for at least one of the five target conditions. Depression accounted for 52% of treated cases, followed by epilepsy (21%), substance use disorders (13%), psychosis (9%), and suicide risk (5%). Seventy-eight percent of clients were female, and 72% were aged between 18 and 49 years.

Fidelity to clinical protocols improved over time. By month 4 of implementation, 86% of PHCs demonstrated correct use of screening tools and treatment guidelines as documented in supervisory checklists (Table 5). WhatsApp supervision logs corroborated these trends, showing a decline in basic diagnostic queries and an increase in more complex case discussions over the implementation period, which supervisors interpreted as evidence of growing provider competence. Providers reported that structured job aids and ready access to specialist advice made them more comfortable managing conditions such as depression and epilepsy within PHC workflows.

**Table 5:** Service delivery coverage and fidelity summary.

Indicator	Baseline	Endline	Data source	Interpretation caveat
PHCs participating	NA	57	Programme records	Administrative scope, not outcome measure
Clients screened (12 months)	Low thousands	64 107	Facility registers	Increased detection not equivalent to treatment quality
Clients initiated on treatment	<50% of screened	9138	Facility registers	No counterfactual available
Referral completion rate (%)	<40	∼60	Referral logs	Influenced by transport costs and stigma
PHCs receiving regular supervision (%)	<50	>75	Supervision checklists	Fidelity varies by district
Protocol adherence (%)	∼55–60	86 (month 4)	Fidelity audits	Documentation quality variable
Weekly case review (%)	13	92.3	Supervision checklists	Self-reported compliance
Client satisfaction (%)	NA	82	Exit surveys	Single-point assessment

Indicators describe scale-up trends rather than causal impacts. All values are derived from routine facility data and self-reported survey instruments.

Client satisfaction remained high throughout the implementation period. In exit surveys, the majority of respondents reported respectful treatment, willingness to recommend services, and perceived symptom improvement. Qualitative data from focus group discussions suggested that communities increasingly viewed PHCs as legitimate sites for mental health care, with providers noting a visible shift in attitudes and reductions in overt stigma among service users.

Frontline providers described these shifts in community expectations:

‘*Initially many patients were surprized to see mental health services in the health centre and worried about stigma. As more people received care and improved, others began to come on their own. Stigma has not disappeared, but mental health services are gradually becoming part of what the community expects from the facility.’*

***—Frontline provider***

However, challenges persisted, particularly in relation to psychosis and suicide risk. Many providers reported hesitancy in initiating antipsychotic treatment without direct specialist input, and referrals to tertiary centres were logistically difficult for some clients due to transportation costs and caregiving responsibilities. These factors contributed to lower confidence scores and a greater reliance on referrals for severe conditions compared with common mental disorders and epilepsy. Nonetheless, the inclusion of MNS care into routine PHC workflows, supported by digital and in-person supervision, represented a foundational shift in service delivery norms in Lagos State.

### Health information systems

Prior to the TTS intervention, mental health indicators were not captured in any routine monitoring or reporting systems in Lagos State; PHCs lacked standard registers for MNS conditions, and district-level managers had no visibility into mental health service coverage or performance. MeHPriC addressed this gap by introducing standardized mental health registers, supervisory fidelity checklists, and simple facility-level dashboards tailored to PHC workflows. Each participating facility was equipped with registers capturing client demographics, diagnosis, treatment plan, and follow-up outcomes, and these data were aggregated monthly and submitted through existing reporting lines to district supervisors.

Efforts were also made to integrate mental health indicators into the District Health Information System 2, Nigeria’s national health data platform. By the end of 2018, a set of mental health indicators (including numbers screened, diagnosed, and treated) had been piloted in 12 facilities, with state-level managers reporting that full rollout was contingent on national approval of the indicator set. Data quality audits revealed that reporting completeness improved from 61% to 89% over a 6-month period, although underreporting and inconsistent coding persisted in some facilities, particularly during early implementation.

Supervisors and facility managers emphasized how routine monitoring strengthened accountability and supported local problem-solving:

*‘Before the registers, it was hard to know how many cases were being seen or where gaps existed. Now we review the numbers during supervision and use them to plan visits and provide targeted support. Reporting is still improving, but the data have changed how we manage services.’*

***—District supervisor***

Nonetheless, incomplete integration of mental health indicators into the national health data platform and the absence of direct links to performance-based financing mechanisms limited the extent to which data could be used to drive broader system-level improvements.

### Cross-cutting enablers and constraints

Across domains, several cross-cutting factors shaped implementation trajectories. Enablers included political commitment at senior levels, alignment with existing administrative structures, availability of dedicated supervisory personnel, and the flexibility afforded by digital supervision platforms. Trust-building between frontline providers and supervisors supported uptake of new practices.

Constraints included procurement delays, variability in facility readiness, competing service priorities, and continued dependence on individual champions to sustain momentum. Although institutional embedding progressed across multiple domains, the depth and pace of integration varied by function and administrative level.

## Discussion

### Principal findings

This study examined the institutional embedding of a government-led scale-up of task-shifted mental health services in Lagos State during the TTS phase of the MeHPriC initiative. Rather than demonstrating system-wide transformation, the findings are consistent with incremental strengthening of the mental health subsystem across governance, supervision, medicines procurement, financing, service delivery, and information systems. Formalization of a Mental Health Desk within the Ministry of Health, absorption of supervisory psychiatrists into government payroll structures, integration of essential psychotropic medicines into routine procurement channels, and incorporation of mental health indicators into facility registers can be interpreted as evidence of movement toward stronger institutional ownership within the public sector, although this ownership remains partial and evolving (World Health Organization 2007). Variability in execution, continued dependence on individual champions, procurement delays, and fiscal uncertainty underscore the uneven nature of embedding, consistent with observations from other LMIC health system strengthening efforts (Sheikh et al. 2011).

The supervision model, combining district psychiatrists with digitally mediated WhatsApp support, appears to have supported provider confidence, continuity of clinical decision-making, and distributed learning across facilities. Task shifting was operationalized primarily among nonphysician cadres, whereas medical officers functioned within mixed-cadre teams. These patterns align with evidence that structured supervision is central to maintaining quality and safety in task-shared mental health services (World Health Organization 2008, Bolton et al. 2023). However, the findings should be interpreted cautiously. The study documents institutional configurations and implementation processes rather than causal effects or system-wide spillover beyond the mental health domain, reflecting methodological limits inherent in descriptive case study designs (Yin 2018).

### Positioning within the scale-up and task-shifting literature

The Lagos experience complements and extends existing evidence on mental health integration in LMICs. Large multicountry initiatives such as PRIME and EMERALD illustrated that task-shifted models can achieve clinical feasibility, provider acceptability, and early service utilization gains when embedded within primary care platforms (Lund et al. 2012, Semrau et al. 2015). Comparative analyses drawing on these programmes have further documented the obstacles and synergies encountered when moving from demonstration pilots toward system-embedded delivery across diverse national contexts (Petersen et al. 2019). Nonetheless, much of this literature remains focused on pilot implementation and short-term outcomes, with fewer analyses documenting how governance, financing, and supply systems evolve as programmes transition from externally supported pilots toward government ownership.

The present findings contribute to this gap by documenting institutional trajectories across multiple system domains over several years. The absorption of supervisory psychiatrists into state employment structures and the incorporation of psychotropic medicines into routine procurement systems respond directly to widely cited sustainability bottlenecks in task-shifted mental health programmes (Rathod et al. 2017). Analyses drawing specifically on the MeHPriC context have further identified the multilevel adaptive mechanisms and contextual dynamics that shape the sustainability of mental health integration within primary care (Adewuya et al. 2025b). Moreover, the use of digitally mediated supervision resonates with emerging evidence that mobile platforms can extend specialist reach, reinforce peer learning, and reduce professional isolation in constrained health systems (Bolton et al. 2023, Ndetei et al. 2023).

Comparative examples from other task-shifting programmes offer useful points of reference. The Friendship Bench in Zimbabwe, for instance, demonstrated that lay health workers could deliver structured psychotherapy for depression within primary care settings, achieving significant symptom reduction with high acceptability (Chibanda et al. 2015). The MeHPriC experience shares certain features with these initiatives, particularly the reliance on structured supervision and the integration of mental health into existing health facility workflows. Where it differs is in its emphasis on documenting the institutional and governance processes through which scale-up was operationalized, rather than focusing primarily on clinical outcomes or acceptability. This institutional focus is itself a contribution to the literature, given that sustainability bottlenecks in task-shifted programmes are more frequently attributed to governance and financing failures than to clinical deficits.

At the same time, the findings align with broader debates regarding the limits of disease-specific programmes as engines of health system change. Analyses of vertically funded HIV initiatives illustrate that targeted investments may strengthen selected system functions without necessarily generating cross-sectoral spillover or durable institutional transformation (Sheikh et al. 2011). In this case, institutional embedding was largely confined to the mental health subsystem, with limited evidence of diffusion into unrelated service areas. This reinforces the analytic distinction between programme-level system strengthening and system-wide reform, a distinction that remains under-theorized in mental health policy discourse (World Health Organization 2007).

### Selected implications for government-led scale-up

Several practical implications emerge for policymakers and implementers. Governance anchoring appears central to sustaining scale-up beyond project cycles. The formal establishment of a Mental Health Desk facilitated coordination across fragmented administrative units and enhanced the visibility of mental health within routine planning processes, consistent with governance theories emphasizing institutional legitimacy and bureaucratic alignment (Sheikh et al. 2011). However, continued reliance on individual champions suggests that organizational routines and incentive structures remain incompletely institutionalized.

The supervision architecture illustrates the potential of combining hierarchical clinical oversight with flexible digital platforms. WhatsApp-mediated supervision enabled timely consultation and iterative learning while minimizing additional infrastructure costs. Similar digitally supported supervision models have demonstrated feasibility in other LMIC mental health programmes, although questions remain regarding governance, data protection, and clinical accountability (Bolton et al. 2023, Ndetei et al. 2023).

Integration into routine procurement and budgeting systems represents a necessary but insufficient condition for sustainability. Although medicine availability and budget visibility improved, operational inefficiencies and fiscal volatility persisted, echoing prior findings that institutionalization exposes programmes to broader public-sector constraints rather than insulating them from them (Rathod et al. 2017). These dynamics highlight the importance of adaptive management and continuous negotiation within bureaucratic systems.

Finally, task shifting remains contingent on supervision intensity, role clarity, and workload management. Although nonphysician providers assumed frontline responsibilities consistent with mhGAP guidance (World Health Organization 2008), boundaries between cadres require continual recalibration, particularly for conditions such as epilepsy that straddle neurological and psychiatric domains (Singh and Sander 2020).

### Limitations and boundary conditions

Several limitations warrant careful interpretation of these findings. The retrospective, single-site, descriptive–explanatory case study design constrains the inferences that can be drawn: reliance on routine administrative data, stakeholder accounts, and programme records introduces potential reporting bias and incomplete documentation, whereas the absence of a comparison group precludes formal causal inference regarding observed institutional changes. Findings should therefore be read as plausible associations and implementation processes rather than definitive evidence of effect.

Generalizability is bounded in important ways. Lagos State’s comparatively strong administrative capacity, fiscal resources, and concentrated urban infrastructure may limit transferability to settings with weaker governance foundations or more dispersed service delivery systems, a consideration that applies particularly to the digital supervision model, whose feasibility may be contingent on connectivity and institutional infrastructure not uniformly available across Nigerian states or comparable LMIC contexts.

Analytic constraints also deserve acknowledgement. Disentangling the relative contributions of project facilitation, senior political commitment, and ambient health sector dynamics remains inherently difficult in embedded implementation research, where the programme and the system it seeks to change are not cleanly separable (Sheikh et al. 2011). Although triangulation across multiple data sources strengthens credibility, the influence of researcher positionality, given the research team's involvement in programme implementation, cannot be entirely discounted, notwithstanding the reflexive practices described in Section 2.7.

Finally, the observation window, although extended to December 2019, may be insufficient to assess whether institutional arrangements prove durable under changing political or fiscal conditions beyond the study period. The digital supervision model was similarly assessed through descriptive indicators and participant perceptions rather than formal effectiveness evaluation, leaving questions about long-term clinical governance, data protection, and accountability structures inadequately resolved: limitations that future prospective research should address directly.

### Policy and research implications

From a policy perspective, the findings suggest that government-embedded scale-up strategies should prioritize early alignment with governance, financing, supervision, and procurement systems rather than operating parallel delivery structures, consistent with health systems strengthening frameworks (World Health Organization 2007). Digital supervision platforms may offer scalable mechanisms for extending specialist capacity, although regulatory oversight remains essential.

For research, the study underscores the value of longitudinal, system-embedded case studies that move beyond pilot efficacy toward institutional dynamics during scale-up (Lund et al. 2012, Semrau et al. 2015). Comparative designs across jurisdictions, economic evaluations of digital supervision models, and longer-term assessments of fiscal sustainability would strengthen the evidence base. Greater integration of routine health information systems may also enable more robust monitoring of equity and quality over time.

### Conclusion

This study documents how a government-led scale-up of task-shifted mental health services was progressively embedded within routine public-sector arrangements in Lagos State, Nigeria. Rather than evidencing system-wide transformation, the findings illustrate incremental strengthening of governance coordination, supervisory capacity, medicines procurement, budgeting processes, and routine data use within the mental health subsystem. These patterns align with health systems perspectives that emphasize institutional alignment and administrative integration as prerequisites for sustainable service expansion (World Health Organization 2007, Sheikh et al. 2011).

The experience reinforces the feasibility of extending mhGAP-aligned care through nonspecialist providers when supported by structured supervision and organizational anchoring (World Health Organization 2008). At the same time, persistent operational constraints, fiscal uncertainty, and dependence on leadership continuity underscore the fragility of institutional gains and the need for adaptive management during scale-up. Digital supervision platforms appear to offer practical mechanisms for extending specialist reach in resource-constrained settings, although governance and accountability considerations remain salient (Bolton et al. 2023, Ndetei et al. 2023).

More broadly, the study contributes empirically to debates on how vertically focused mental health programmes can transition toward durable public-sector ownership within the mental health subsystem without assuming automatic spillover into broader health system reform. Continued investment in longitudinal, system-embedded research will be essential for understanding how institutional configurations evolve over time and under changing political and fiscal conditions (Lund et al. 2012, Semrau et al. 2015).

## Supplementary Material

czag033_Supplementary_Data

## Data Availability

The data underlying this article will be shared on reasonable request to the corresponding author.

## Reflexivity statement

The authorship team comprises seven Nigerian researchers (five males and two females) with extensive experience in mental health policy, clinical practice, and health systems implementation in Nigeria. The group includes senior psychiatrists (with professorial and consultant-level expertise) and social workers, spanning multiple levels of seniority from established academics to mid-career professionals. All authors are based in Lagos, Nigeria, and have longstanding collaborative relationships with the Lagos State Ministry of Health, Primary Health Care Board, and other local stakeholders, ensuring deep contextual knowledge and direct engagement with the study setting. This composition supports strong local ownership of the research question, design, and interpretation, while reflecting gender diversity within a predominantly Nigerian team.
