# Supplementary material for: Government-led scale-up of task-shifted mental health services in Lagos State, Nigeria: a mixed-methods descriptive–explanatory case study
Source: Health Policy Plan. 2026 Mar 10;41(5):785–97. doi: 10.1093/heapol/czag033 (PMC13187677; doi:10.1093/heapol/czag033)
Supplement: czag033_Supplementary_Data [file czag033_supplementary_data.docx]

**Supplementary Table S1**

Data sources, indicators, sample sizes, and analytical domains used in the Transition-to-Scale evaluation



| **Data source** | **Data type** | **Primary indicators** | **Sample/**  **Coverage** | **Time period** | **Analytical domain** |
| --- | --- | --- | --- | --- | --- |
| Facility service registers (PHCs and GHs) | Quantitative | Screened; diagnosed; treated; follow-up; referral | 57 PHCs; 5 GHs; 64,107 screened | Mid-2017 to Dec 2019 | Service delivery; HIS |
| PHC monthly summary reports | Quantitative | Aggregated MNS indicators; referrals | 57 PHCs | Mid-2017 to Dec 2019 | Health information systems |
| Supervision checklists | Quantitative | Visit frequency; cases reviewed; protocol adherence | 57 PHCs; repeated audits | Mid-2017 to Dec 2019 | Workforce; governance |
| WhatsApp supervision logs | Quantitative + qualitative | Clinical queries (avg 34/district/month); response time (<4 hrs); query themes | 5 district groups; >500 providers | Mid-2017 to Dec 2019 | Workforce; service delivery |
| Training registers | Quantitative | Cadre; completion rate (>95%) | 890 health workers | Mid-2017 to mid-2019 | Workforce |
| Medicine stock audits | Quantitative | Availability of 6 psychotropics; stockouts | 57 PHCs; baseline + follow-up | Baseline 2016; follow-up 2018 | Medicines |
| Budget documents | Documentary | Allocations; line items; expenditure | State-level | 2016–2019 | Financing; governance |
| MTSS documents | Documentary | Mental health in planning frameworks | State-level | 2017–2019 | Governance; financing |
| Policy circulars | Documentary | MH Desk establishment; EML revision | State-level; >25 documents | 2017–2019 | Governance |
| Stakeholders Council minutes | Documentary | Attendance; decisions | 6 meetings | 2017–2019 | Governance |
| KIIs (policy level) | Qualitative | Governance; sustainability; barriers | n = 18 | 2018–2019 | Governance; financing |
| KIIs (facility/district) | Qualitative | Supervision; medicines; workflow | n = 22 | 2018–2019 | Workforce; medicines |
| FGDs (PHC providers) | Qualitative | Task shifting; confidence; supervision | 6 FGDs; 8–10 participants each | 2018 | Workforce; service delivery |
| Provider survey | Quantitative | Knowledge; confidence; supervision experience | n = 726 (82% response rate) | Mid-2018 | Workforce; supervision |
| Client exit survey | Quantitative + qualitative | Satisfaction; acceptability; improvement | n = 2,380 | 2018 | Service delivery |
| DHIS2 pilot reports | Quantitative + documentary | MH indicator integration; completeness (61% to 89%) | 12 pilot facilities | 2018–2019 | Health information systems |

**Note**: KIIs = key informant interviews; FGDs = focus group discussions; GHs = general hospitals; MNS = mental, neurological, and substance use; HIS = health information systems.
